# Medical oncology care amidst the COVID-19 pandemic at the National University Hospital in the Philippines

**DOI:** 10.3332/ecancer.2020.1066

**Published:** 2020-06-30

**Authors:** Marvin Jonne L Mendoza, Harold Nathan C Tan, Aylmer Rex B Hernandez, Brylle Caesar A Dala, Danielle Benedict L Sacdalan, Dennis L Sacdalan, Gerardo H Cornelio, Jorge G Ignacio

**Affiliations:** 1 Division of Medical Oncology, Department of Medicine, University of the Philippines—Philippine General Hospital, Manila, Philippines; 2 Department of Pharmacology and Toxicology, College of Medicine, University of the Philippines Manila, Philippines; 3 Chair, Cancer Institute and Division of Medical Oncology, Department of Medicine, University of the Philippines—Philippine General Hospital, Manila, Philippines; a https://orcid.org/0000-0002-0191-4766; b https://orcid.org/0000-0003-3432-3062; c https://orcid.org/0000-0001-6151-6957; d https://orcid.org/0000-0003-0866-180X; e https://orcid.org/0000-0002-7400-8078; f https://orcid.org/0000-0001-6617-9363

**Keywords:** care delivery, COVID-19, medical oncology, Philippines, Philippine General Hospital

## Abstract

COVID-19 has abruptly and radically changed the landscape of cancer care delivery throughout the world, including the Philippines. The Philippine General Hospital is the academic hospital of the University of the Philippines. Its cancer centre is a primary referral centre that takes care of Filipinos—many resource-constrained—that are burdened by malignancy. As the global pandemic challenges healthcare delivery, centres are forced to rethink how to care for their patients. This paper discusses how a national, academic, referral cancer institute in a low–middle income country is trying to meet the challenges of COVID-19.

## The advent of COVID-19

COVID-19 has abruptly and radically changed the landscape of cancer care delivery in the Philippine General Hospital Cancer Institute. In response to the surge of cases in the country, the Philippine General Hospital (PGH), the academic hospital of the University of the Philippines, was designated by the Philippine Department of Health as a COVID-19 referral centre [1]. This meant that the 1,500-bed multidisciplinary academic hospital would allocate 130 beds for the treatment of COVID-19 positive patients, with the possibility of upscaling operations as resources allowed.

Non-emergency clinics, elective surgeries and non-COVID-19 admissions were suspended until further notice. All subspecialty internal medicine fellows-in-training—including medical oncologists—were tasked to join the pool of healthcare workers at the frontline of the COVID-19 response. As such, the Cancer Institute discontinued its operations for one week. Cognizant of the detrimental effect of the cessation of oncologic services to cancer patients, the oncology team used this time of closure as an opportunity to assess the unmet needs of the cancer patients amidst the pandemic, evaluate the feasibility of resuming its operations, and plan ways to adapt to the new situation.

## Cancer care in the pre-pandemic era

Before the COVID-19 pandemic, the Medical Oncology Clinic of the PGH Cancer Institute has served as a referral centre for indigent cancer patients from all over the country. The Division of Medical Oncology consists of 14 medical oncology fellows and 10 consultant staff. On a regular day, the outpatient clinic caters to 150–200 cancer patients for consultations and outpatient treatment. Meanwhile, 20 beds are available at the Cancer Institute for inpatient chemotherapy and other oncologic care interventions.

## New normal, old problems

The high volume of patients needing care meant long queues at the outpatient clinic and prolonged waiting times for ward admissions before COVID-19. As a result of the changes undertaken by the hospital to meet the challenges of the current pandemic, many patients became at risk of not receiving adequate oncologic care.

The COVID-19 pandemic has caused several issues to arise. First among these is safety, both for patients and healthcare workers. Cancer patients have an increased risk for serious complications arising from COVID-19 infection, including a higher probability of death. Patients with lung cancer and hematologic malignancies are particularly prone to developing severe events [2, 3]. The threat posed by COVID-19 on the health of patients must be weighed against the likelihood of tumour progression and its associated morbidity resulting from treatment delays. A careful balance between protecting cancer patients from contracting the virus and proper management of the malignancy needs to be achieved.

The safety and overall well-being of the medical staff also needs to be addressed. This goes beyond simply providing adequate personal protective equipment. In our institution, physicians who have been assigned to units with COVID-19 patients continue to work unless they present with symptoms related to the infection or have had a positive test for SARS-CoV-2. Those assigned to the COVID-19 areas go on duty for 24 hours two to three times in a week. This translates to a 100% increase in their work hours. Prolonged periods of exposure to active cases of COVID-19 coupled with increased workload can cause psychological distress among health professionals. Furthermore, allowing physicians to work in both COVID-19 and cancer wards carries a significant risk of cross-contamination by undocumented, asymptomatic medical personnel [4, 5]. Staff issues need to be addressed to ensure adequate cancer care for patients during these difficult times.

Another important issue is the resource constraints in our institution. Utilisation of ward beds, medications, blood products, staff, and basic medical supplies may conflict with delivery of medical services for patients with COVID-19 [4]. Without proper protocols in place, the increasing demands of COVID-19 would hinder the capacity of the hospital to address the needs of cancer patients. Since medical care in the Philippines is largely paid for out-of-pocket, indigent patients obtain their medicines through the hospital’s medical social services, charities, or non-governmental organisations. Unfortunately, due to the current pandemic these traditional sources have switched their priorities to supporting COVID-19 directed efforts or have stopped operations temporarily. Resource allocation needs to be carefully considered to provide the best possible care to our cancer patients.

## Adapting to the changes

To meet the challenges imposed by COVID-19, the Cancer Institute devised a comprehensive approach to facilitate care delivery. This pathway was implemented following re-opening of the Cancer Institute last April 1, 2020. The strategies developed to achieve these goals are summarised in Table 1.

To reduce the number of clinic visits and the possibility of exposure of patients and staff to COVID-19 cases, a hotline for medical oncology patients was created to triage concerns and to determine if a face-to-face consultation was necessary. Prioritisation for delivery of systemic cancer treatment was based on the potential treatment benefit and therapeutic intent [3, 6]. Treatment schedules were revised to minimise the possibility of patient exposure to COVID-19. Shared decisions to modify treatment were made with each individual patient. Whenever possible, intravenous regimens were shifted to oral or subcutaneous therapies, and chemotherapy cycle intervals were slightly prolonged to reduce the frequency of clinic visits without significantly compromising effectiveness.

The Cancer Institute scaled down its operations to allow sufficient time for proper patient screening and to comply with social distancing guidelines. When it re-opened in April 2020, patient visits to the Medical Oncology Clinic went down by 90%. Each day, a maximum of 20 patients were accommodated at the outpatient chemotherapy unit, based on the prioritisation scheme stated above. All appointments were pre-scheduled for old patients using the hotline while acceptance of new patients and referrals was limited to avoid overcrowding in the facility.

Patients requiring hospital admission are currently being accommodated, with careful adjustment of inpatient bed capacity based on the evaluation of the management team. Patients who cannot be seen at the Cancer Institute are endorsed to medical oncologists residing within their own localities, especially those needing urgent treatment but restricted from traveling to Manila, where the Cancer Institute is located, due to the island-wide community quarantine.

**Figure 1.**
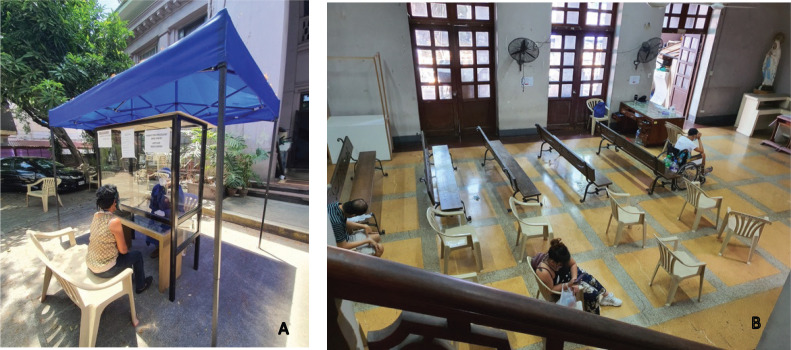
Infection control measures are implemented through: (A) centralised screening for COVID-19-related symptoms upon entry of patients and (B) strict social distancing protocols at the waiting area.

**Figure 2.**
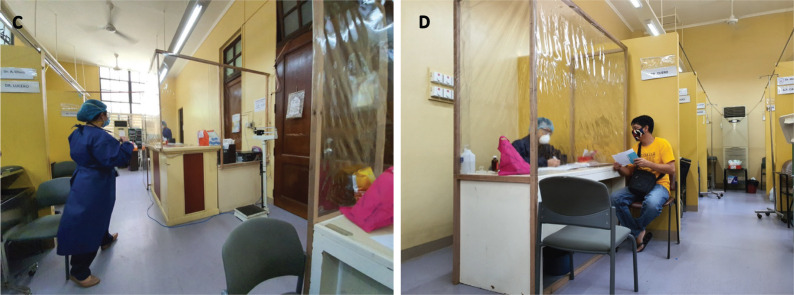
(A) Installation of plastic shields at the outpatient clinic to serve as protective barriers between the patient and healthcare worker and (B) Wearing of proper protective measures both by the healthcare worker (N95 face mask, goggles or face shield, hair cover, disposable gown and surgical gloves) and the patient (surgical mask).

Other infection control measures have been implemented. First, the entry and exit points of cancer patients are redirected away from the COVID-19 areas, reducing the risk of cancer patients interacting with COVID-19 patients. Upon arrival at the Cancer Institute, each patient undergoes centralised screening for COVID-related symptoms (Figure 1). If a patient presents with symptoms, they are promptly directed to the COVID triage for evaluation and admission. Patients and their caregivers are always given face masks to wear inside the premises. Cough etiquette, hand hygiene and social distancing protocols are strictly enforced. The outpatient clinic and chemotherapy room have been redesigned with plastic shields (Figure 2) to serve as protective barriers between doctors and patients. These areas are also disinfected daily after use.

To address other medical staff issues, the following measures have been instituted. The fellows-in-training are divided into COVID and non-COVID teams. The latter team is responsible for providing oncologic services at the Cancer Institute. Members of each team are not allowed to interact with each other. This segregation minimises the risk of cross-contamination between teams. Use of appropriate PPEs, depending on exposure risk, and strict adherence to infection control measures, are enforced [4, 5]. As the pandemic takes a toll on the psychological well-being of the medical staff, the Division of Medical Oncology has enlisted the help of the Department of Psychiatry in creating a mental health and transformational change programme, and in conducting regular psychosocial processing. The medical staff are given the opportunity to process their fears and anxieties, helping them develop healthy coping strategies to deal with their emotions.

In terms of resource constraints, the Cancer Institute has sought the help of non-governmental organisations to ensure that the resources needed by cancer patients and the medical staff are met. Through their aid, sufficient medical supplies and equipment have been obtained to protect the workforce. Patient access programmes for systemic treatment are facilitated through communication with pharmaceutical companies and non-governmental organisations. To mitigate disruption in the supply chain, a centralised inventory of medical consumables is coordinated with the Central Supply Unit. Because many elective diagnostic tests cannot be done in PGH, pre-chemotherapy laboratories and other pertinent imaging studies are performed in other healthcare facilities prior to admission or consultation.

## Moving forward

With the easing of the enhanced community quarantine and with continuous evaluation of the pathways, processes and guidelines, the Medical Oncology Clinic is slowly returning to its normal operations. Following recommendations of the PGH hospital infection control unit, asymptomatic cancer patients who are scheduled for chemotherapy are to have the RT-PCR SARS-CoV-2 of nasopharyngeal (NP) or oropharyngeal (OP) samples in their diagnostics as part of the pre-chemotherapy evaluation. The medical oncology fellows have received training in proper swabbing technique. NP or OP samples are sent to the molecular research laboratory of the Philippine General Hospital for testing. Confirmed positives are promptly transferred to COVID-19 areas for appropriate care. This system has allowed the Cancer Institute to continue operations while mitigating the risk of transmission of COVID-19 to vulnerable cancer patients and healthcare staff. Up until now, this system has allowed the detection and prompt management of otherwise asymptomatic cancer patients with COVID-19. Equally important, no clinic staff members or healthcare workers have contracted COVID-19.

Realising that this is a work in progress, quality control measures are conducted through feedback and satisfaction surveys from the patients. Presently, the Medical Oncology Clinic has been accommodating more patients—doubling the number seen per day compared to when it re-opened in April. Additionally, duties in the COVID units have been decreased as PGH begins resuming non-COVID services. This has allowed the medical oncology staff to re-focus on the care of cancer patients. Our experience is certainly different from those of other centres and there is no singular approach to addressing this challenge [7]. Regular team meetings allow insight from the healthcare team to be discussed and changes implemented. Prospective studies are planned to follow short- and long-term outcomes to evaluate the impact of the situation on patient outcomes.

## Conclusion

Providing cancer care during the COVID-19 pandemic is a challenge. The safety of patients and healthcare providers have been a central tenet of policy decision-making at the PGH Cancer Institute. Through the concerted efforts of all stakeholders, we have been gradually resuming the care of our cancer patients.

## Ethical considerations

This paper is a narrative description of healthcare innovations implemented at the Philippine General Hospital Cancer Institute during this unprecedent time of the COVID-19 pandemic. It does not involve human participants, identifiable human tissue, biological samples or data. It is exempted from ethical review based on the National Ethical Guidelines for Health and Health-Related Research of the Republic of the Philippines published in 2017.

## Conflicts of interest statement

The authors declare no conflicts of interest in relation to this work

## Funding sources

The authors did not receive external funding for this work

## Figures and Tables

**Table 1.** Strategies adapted by the Cancer Institute during the COVID-19 pandemic.

**Promoting patient safety**
Creation of a patient hotline for triaging cancer patients Prioritisation of patients for systemic cancer treatment Pre-scheduling and pre-screening of all patients for outpatient consult and admission Coordination of referrals to local oncologists for patients with travel restrictions
**Promoting health and safety of healthcare workers**
Appropriate use of personal protective equipment (PPE) Physical and procedural restructuring of the outpatient clinic Strict screening of all persons entering the Cancer Institute premises Observance of proper hand hygiene and social distancing Segregation of healthcare personnel working in the institute from those working in the COVID-19 areas Provision of psychosocial support to healthcare personnel
**Addressing resource constraints**
Linkages with non-governmental organisations to ensure adequate supply of essential resources Development of a centralised inventory system of medical consumables
